# Sustainable waiting time reductions after introducing the STAT model for access and triage: 12-month follow up of a stepped wedge cluster randomised controlled trial

**DOI:** 10.1186/s12913-020-05824-z

**Published:** 2020-10-21

**Authors:** Katherine E. Harding, David A. Snowdon, Luke Prendergast, Annie K. Lewis, Bridie Kent, Sandy F. Leggat, Nicholas F. Taylor

**Affiliations:** 1 Allied Health Clinical Rsearch Office, Eastern Health, Level 2/5 Arnold Street, Box Hill, VIC 3128 Australia; 2 La Trobe University, Kingsbury Drive, Bundoora, VIC 3086 Australia; 3 Drake Circus, Plymouth University, Plymouth, Devon PL4 8AA UK

**Keywords:** Outpatients, Community health, Waiting lists, Triage, Appointments and schedules

## Abstract

**Background:**

Timely access is a challenge for providers of outpatient and community-based health services, as seen by the often lengthy waiting lists to manage demand. The Specific Timely Appointments for Triage (STAT) model, an alternative approach for managing access and triage, reduced waiting time by 34% in a stepped wedge cluster randomised controlled trial involving 8 services and more than 3000 participants. Follow up periods ranged from 3 to 10 months across the participating services in accordance with the stepped wedge design. This study aimed to determine whether outcomes were sustained for a full 12 months after implementation of the STAT model at each site.

**Methods:**

Routinely collected service data were obtained for a total of 12 months following implementation of the STAT model at each of the 8 services that participated in a stepped wedge cluster randomised controlled trial. The primary outcome was time to first appointment. Secondary outcomes included non-attendance rates, time to second appointment and service use over 12 weeks. Outcomes were compared to pre-intervention data from the original trial, modelled using generalised linear mixed effects models accounting for clustering of sites.

**Results:**

A 29% reduction in waiting time could be attributed to STAT over 12 months, compared to 34% in the original trial. A reduction in variability in waiting time was sustained. There were no significant changes in time to second appointment or in the number of missed appointments in the extended follow up period.

**Conclusions:**

STAT is an effective strategy for reducing waiting time in community-based outpatient services. At 12 months, small reductions in the overall effect are apparent, but reductions in variability are sustained, suggesting that people who previously waited the longest benefit most from the STAT model.

**Trial registration:**

This is a 12-month follow up of a stepped wedge cluster randomised controlled trial that was registered with the Australia and New Zealand Clinical Trials Registry (ACTRN12615001016527).

## Background

As demand for health care services increases, providers of health services often face challenges in ensuring timely access [1, 2]. Adding incoming referrals to the end of a waiting list is often used as a default strategy in managing demand, sometimes supplemented with triage systems of varying complexity [3, 4]. Excessive wait times can lead to poorer outcomes for patients and higher costs for health services as resources are diverted from clinical care to administrative processes associated with managing the waiting lists [5, 6]. When waiting lists become unacceptably long, strategies such as a temporary increase in supply may be implemented, but do not address underlying imbalances between supply and demand. This leads to the recurrence of waiting lists once the resources are withdrawn [7]. Fundamental changes are needed to balance the distribution of resources with the demand for the service for timely access to be maintained.

The Specific Timely Appointments for Triage (STAT) model of access and triage has demonstrated effectiveness in reducing waiting times for community-based outpatient services [8]. The primary principle of STAT is that the rate of service demand is calculated, and the corresponding number of new appointments required each week to meet this demand is protected in clinician schedules [9, 10]. All patients receive access to a prompt, face to face assessment, but the assessing clinician then makes prioritisation decisions about the ongoing allocation of services based on the needs of the patient, in the context of overall demand for the service. This is complemented by a short-term, targeted intervention to reduce or clear the backlog of waiting patients prior to the introduction of protected appointments for new patients [9, 11].

In a stepped wedged randomised controlled trial, STAT was progressively introduced across 8 community-based outpatient services [9]. A 34% reduction in waiting time was attributed to the intervention. A reduction in variability in waiting time was also observed, with fewer patients waiting long periods for a first appointment following the intervention [8]. The reductions in waiting time and variability achieved in this trial were similar to pilot trials of the STAT intervention conducted in community rehabilitation and outpatient physiotherapy settings [10, 12].

A criticism of many approaches to reduce waiting time is that they result in short-term changes without sustained benefits. Without demonstration of sustainability of STAT and other approaches, it is difficult to know whether early benefits are due to a short-term increase in supply or due to a change in process. The period of post intervention data collection in the stepped wedge trial ranged from 3 to 10 months after implementation of STAT at each site (depending on the timing of intervention at each site within the stepped wedge design) [9]. The findings of the trial over this period were promising, but there remained an unanswered question about the sustainability of the STAT model over a longer period. This study aimed to determine whether the STAT model was effective in sustaining a reduction in waiting time for community-based outpatient services over a 12-month period following its implementation.

## Method

### Design

**Fig. 1:**
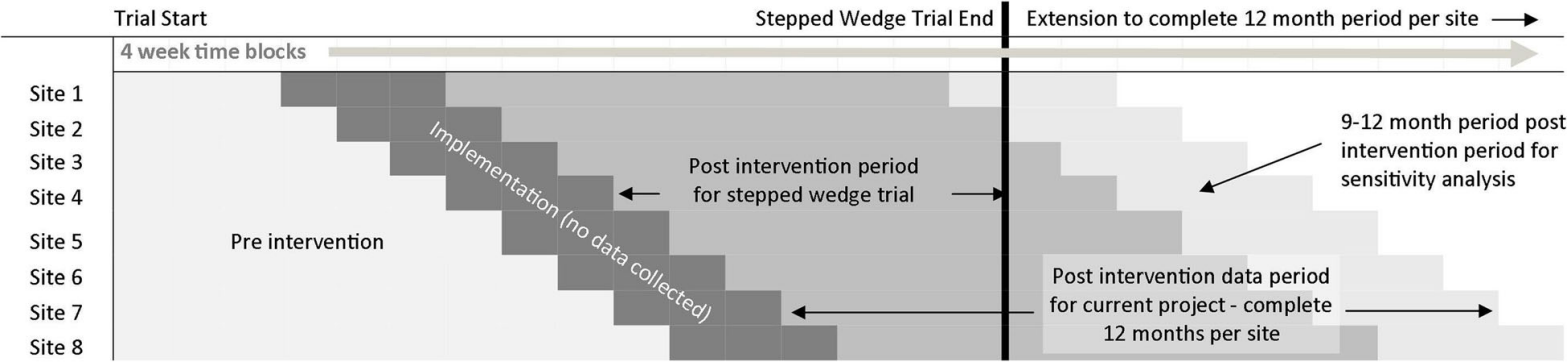
Trial design during pre intervention period (original trial data), first 0–9 months post implementation and 9–12 months post implementation

Routinely collected service data were captured retrospectively from the 8 sites that participated in a stepped wedge cluster randomised controlled trial investigating the effect of STAT on waiting time [8]. The original trial included pre and post data collection periods ranging from 3 to 10 months at each site, depending on the randomised order of intervention at each site. The trial design therefore provided limited insights into the effects of the intervention over time as longer term data (for example 6–10 months post intervention) were only available from a subgroup of the 8 sites that were randomised earlier in the intervention sequence. For the current study, data were collected over an additional period at each site to complete a 12-month period following implementation of STAT at all sites (Fig. 1). The collection of data for the original trial was completed between October 2015 and April 2017. For this study, additional data were collected through to November 2017 to enable a full 12-month follow up period at each site. All patients who had their first appointment at each site within the study period were included in the trial. Data were not collected from new patients who attended their first appointment during the 12 week implementation period at each site.

The original stepped wedge trial was registered with the Australian and New Zealand Clinical Trial Registry (ACTRN12615001016527) [9] and ethical approval was provided by the Human Research Ethics Committee of the health network both for the original trial and this follow up study. Funding was provided by the Australian National Health and Medical Research Council (APP1076777) and the Victorian Department of Health and Human Services. The original study was reported in accordance with the CONSORT statement for reporting of cluster randomised controlled trials. The majority of items in the CONSORT statement are also reported in the current study, but readers are also referred to the protocol and findings of the original study for additional detail [8, 9].

### Setting

This trial was conducted at a metropolitan health network providing care to a population of more than 700,000 people in Melbourne, Australia. Eight sites providing community-based outpatient services within this network participated in the trial. Community-based outpatient services typically consist of allied health, medical and nursing professionals working within multi-disciplinary teams, providing care to people with chronic health conditions, children with developmental disorders or supportive care or rehabilitation services often associated with a hospital admission. The 8 participating sites were purposefully selected from 28 community-based outpatient services within the network that participated in a previous qualitative study exploring factors that contribute to waiting time in these settings [3]. Services were selected on the basis that they provided care over a series of appointments, used waiting lists to manage demand and reported the length of their waiting lists to be reasonably stable over the previous 2 years [9].

### Intervention

Prior to the implementation of STAT, all 8 sites used a waiting list to manage demand for their service. A detailed description of the intervention (STAT) has been published in an online handbook [11]. The key elements of the intervention include: (1) protection of the required number of new appointments to meet demand in clinician schedules; (2) a targeted short-term intervention to reduce the backlog of waiting patients; and (3) redesigning workflow to ensure rapid access to a first appointment, with clinicians then making prioritisation decisions about the patient’s need for ongoing services following their clinical assessment and within the context of demand for their service. The short-term, targeted interventions, used to reduce the backlog of waiting patients prior to the introduction of protected appointments, were individualised for each service and were supported by a small budget [8].

### Outcome measures

The primary outcome measure (waiting time) was the number of days between the service receiving the referral and the patient’s first scheduled appointment. Secondary outcomes included number of days between the first and second scheduled appointment, the total number of appointments over the 12 weeks following the first scheduled appointment, and the number of times the participant did not attend a scheduled appointment during this 12-week period.

All primary and secondary outcome data were routinely collected by the participating services and entered into a health network database. Manual checking of clinician schedules and referrals were completed to verify the accuracy of data obtained from the health network databases.

### Statistical analysis

Outcomes were modelled using generalised linear mixed effects models with a negative binomial dependent variable. A random intercept was included to account for clustering of sites, and a random slope to allow for variation in the effect of intervention across sites, as suggested by Davey et al. [13] Results were reported as incidence rate ratios (IRR). A Gaussian linear mixed effects model was fitted to the log of waiting times as a sensitivity analysis with no notable differences found when comparing with the negative binomial model.

The cohort included in the 12-month follow-up incorporated people who attended the service anytime from 1 day to 12 months after implementation. It was possible that inclusion of data from those who attended early after the implementation of STAT could mask changes in the outcomes that appeared later in the follow-up period. To address this issue, a sensitivity analysis was conducted including the subgroup of patients who received their first visit between 9 and 12 months after implementation. Although it was a smaller sample, this cohort was independent of the participants included in the original stepped wedge trial and was expected to provide a more accurate reflection of the outcomes at each service 12 months after implementation.

Service demand ratio (a ratio of the number of referrals received over a 3-month period early in the trial and a corresponding period in the following year) was calculated to provide an indication of stability of demand at each site.

Previous trials have indicated that the greatest beneficiaries of the STAT model are often the longest waiters, as the model protects against low priority patients languishing on waiting lists for long periods of time, constantly displaced, as higher priority patients are referred. Therefore, in addition to the statistical analysis, observational data of the 90th percentile of waiting time for the three cohorts (pre-intervention, full 12-month follow-up and 9–12 month subgroup) were used as an indicator of ongoing effectiveness of the model for reducing delays for the longest waiters.

## Results

Data were collected from 4358 participants; 1252 in the pre intervention period and 3106 in the post intervention period. The pre and post periods had similar proportions of male and female participants, but those in the pre group were slightly younger on average (mean 40 v 43 years, *p* < 0.01) than the post group (Table 1). The characteristics of the participating sites have been described previously [8]. The demand ratio calculated for the current study suggested more stable demand over the data collection period than was observed in the original trial, with consistent demand noted at 5 services and increases in demand between 10 to 25% in 3 services.

**Table 1:** Patient characteristics

	Pre intervention^a^	12-month follow up	Sensitivity analysis	Significance^b^	
		0 to 12 month post intervention	9 to 12 month post intervention	Pre to 0–12 month	Pre to 9–12 month
**n**	1252	3106	821		
**Sex** *[n(%)]*					
Female	743 (59%)	1896 (61%)	498 (61%)	*p* = 0.30	*p* = 0.58
Male	509 (41%)	1210 (39%)	323 (39%)		
**Age** *[years, mean (SD)]*	43 (30)	40 (30)	39 (30)	*p* < 0.01	*p* < 0.01

^a^Data from original trial. Includes pre intervention periods for the participating sites ranging from 3 to 10 months, consistent with the stepped wedge trial design [8].

^b^Statistical significance calculated using chi square for sex and t-tests for age

### Primary outcome

A 29% reduction in waiting time over the complete 12-month follow up period across all sites was attributable to the intervention (IRR 0.71, 95% CI 0.60 to 0.83, Table 2). Waiting time reduced from a median of 42 days (IQR 19 to 86 days) pre-intervention to 31 days (IQR 14 to 55 days) post-intervention. The reduction in waiting time is less than the reduction of 34% observed at the end of the stepped wedge trial. However, the 95% confidence interval for the IRR in the current study is 0.6 to 0.83, suggesting that such magnitudes of reduction are still possible.

**Table 2:** The effect of STAT on time from referral to first appointment (primary outcome)

	Original trial		12-month follow up	Sensitivity analysis
	Pre-intervention	Post-intervention	0 to 12 month post intervention	9 to 12 month post intervention
**n**	1252	1861	3016	821
**Median (IQR)**	42 (19 to86)	24 (13 to48)	31 (14 to55)	36 (13 to62)
**Mean (SD)**	60.0 (55.2)	35.6 (33.6)	40.0 (35.3)	41.9 (34.7)
**Adj ratio IRR (95% CI)**	Reference	0.66 (0.52 to 0.85)	0.71 (0.60 to 0.83)	0.78 (0.69 to 0.88)

*IRR* incident rate ratio, *Adj ratio* Adjusted ratio indicates that other factors, such as potential confounders, have been included in the model. IRR calculated using a generalised linear mixed effects model

The sensitivity analysis, including patients who received their first appointment 9 to 12 months after implementation (*n* = 821), showed a 22% reduction in waiting time (IRR 0.78, 95% CI 0.69 to 0.88) for patients receiving an initial assessment during this period, compared to those who received their first visit prior to the intervention (Table 2). The 95% confidence interval for the IRR is still significant indicating reductions in time, although in comparison to the 34% reduction observed within the main trial, these reductions are smaller.

Waiting time at the 90th percentile indicated that 90% of patients, who were seen 9 to 12 months after implementation of STAT, were seen within 81 days, compared with 83 days over the full 12-month follow up period and 139 days during the pre-intervention period.

**Fig. 2:**
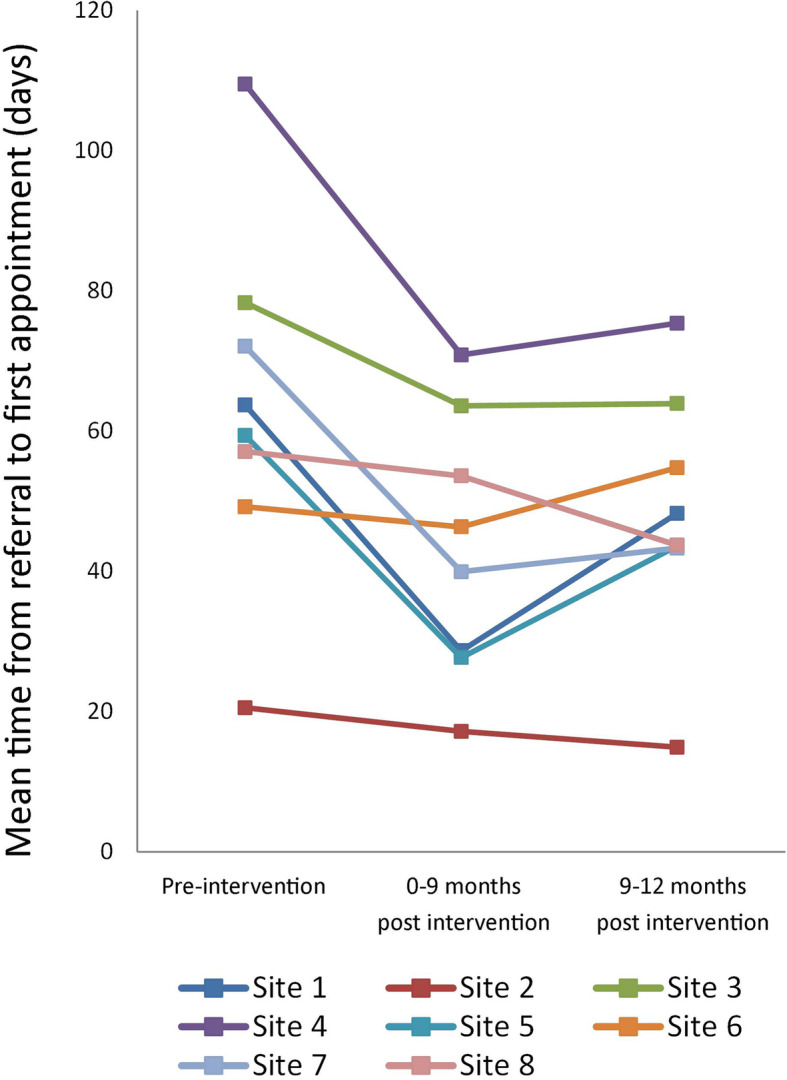
Mean waiting time by study site

The trial was not designed or powered to investigate in-depth site level responses to the intervention. However, post-hoc site level observations at 12 months (Fig. 2) illustrate variability between services, which, on the one hand, may be consistent with the variability expected from sampling from the population of services. On the other hand, although speculative, observations of the services demonstrating increased waiting time during follow up may be partly, but not fully, explained by fluctuations in supply and demand. Three services experienced an increase in demand in the subsequent 12 months (site 4, 5 and 7), but only one of these (site 5) showed a substantial increase in waiting times during the follow up period. However, of the other sites with less success in maintaining reductions in wait time, site 6 had previously reported an unusual spike in demand during the implementation period that may have had an ongoing impact on patient flow through the service, and site 1 experienced a disruption in supply during the 12-month follow up period due to a key senior staff member taking maternity leave. Overall, three of the sites either maintained or further decreased reductions in waiting time at 12 months, and four showed an initial drop in waiting time followed by some deterioration of effect, but did not return to pre-intervention waiting times during the follow up period.

### Secondary outcomes

There were no significant differences in the time from first to second appointment (Table 3). A small (6%) decrease in the total number of appointments within the first 12 weeks after initial assessment from an average of 2.4 to 2.1 appointments was statistically significant over 12-month follow up (IRR 0.94, 95% CI 0.89 to 0.98). Data from the original trial suggested that STAT might have led to a small increase in the number of patients missing appointments. This was not the case over the longer follow up period, where there was an observed, but non-significant, decrease in the number of missed appointments.

**Table 3:** The effect of STAT on secondary outcomes

	Original trial		12-month follow up	Sensitivity analysis
	Pre-intervention	Post intervention	0 to 12 month post intervention	9 to 12 month post intervention
**n**	1252	1861	3016	821
**Appointments missed per patient**, *n*				
*Mean (SD)*	0.4 (0.9)	0.5 (0.7)	0.3 (0.7)	0.3 (0.7)
*Adj Ratio (95% CI)*	*Reference*	IRR: 1.18 (1.04 to 1.35)	IRR: 0.86 (0.69 to 1.07)	IRR: 0.78 (0.63 to 0.97)
**Time from 1st to 2nd appointment**, *days, n*				
*Mean (SD)*	28.8 (20.0)	28.5 (18.5)	29.8 (20.5)	28.3 (19.0)
*Adj Ratio (95% CI)*	*Reference*	IRR: 1.03 (0.98 to 1.09)	IRR: 1.06 (0.94 to 1.19)	IRR: 1.04 (0.96 to 1.14)
**Appointments in first 12 weeks**, *n*				
*Mean (SD)*	2.4 (2.1)	2.1 (1.6)	2.1 (1.6)	2.0 (1.6)
*Adj Ratio (95% CI)*	*Reference*	IRR 0.99 (0.93 to 1.05)	IRR: 0.94 (0.89 to 0.98)	IRR: 0.89 (0.73 to 1.10)

*Adj ratio* Adjusted ratio indicates that other factors, such as potential confounders, have been included in the model

IRR calculated using generalised linear mixed effects models

Considering only the cohort who attended the service 9 to 12 months after the implementation of STAT, observed values of these secondary outcomes were similar to those collected over the full 12-month follow up period. Time from first to second appointment remained unchanged. The small reduction in the number of appointment provided over the first 12 weeks was maintained, although this finding was not significant given the reduced sample size in this subgroup. In contrast, data on missed appointments for this subgroup showed a statistically significant 22% decrease from 0.4 to 0.3 missed appointments per patient over this time period (IRR: 0.78, 0.63 to 0.97, Table 3).

## Discussion

Reductions in waiting time achieved through introduction of the STAT model remained statistically significant at 12-month follow up, although there may have been some small reductions in the gains measured in the original trial. However, patients seen for the first time in the period 9 to12 months following the intervention, still experienced a 22% reduction in waiting time compared to those seen for the first time prior to the intervention controlling for differences between sites.

The findings that significant effects were still seen 9–12 months after intervention is encouraging, but there remains some uncertainty about whether this is a long enough time period to measure sustainability. It needs to be considered whether the effects observed at this time were the direct result of a temporary injection of resources to reduce the backlog, which can be expected to continue to wash out with time. Previous research has shown that short term injections of funding to reduce backlogs are ineffective, with waiting lists beginning to grow back as soon as the resources are withdrawn [14]. Much of this evidence is drawn from efforts to reduce surgical waiting list with temporary increases in activity; there is little to shed light on how long effects of different levels of temporary investment can be expected to last in community outpatient settings. However, the temporary resources provided in this study were modest, and had been expended prior to the beginning of the post implementation period; that is there were no ongoing resources supplied such as continued additional staff or additional clinics. Furthermore, all sites had access to similar levels of resources to assist with backlog reduction, but there was observed to be variation in response over time. This suggests that outcomes at 12 months were not a simple function of the additional investment, but were influenced by service level factors and the other components of the STAT model.

Perhaps the most clinically significant difference between the pre-intervention data and follow up data is the reduction in waiting time for the longest waiters in the sample. Observations of waiting time at the 90th percentile suggest that STAT continued to be very effective in reducing the tail of “long waiters” 12 months after implementation, with no indication of this effect diminishing. Patients given classifications as low priority and waiting excessively long periods for treatment, or not being seen at all, is a well document problem associated with the use of waiting list and priority systems [15]. STAT provides a sustainable way to ensure that these patients are seen in a timely manner without adversely affecting other aspects of patient care.

One explanation for the diminishing effect of the model over time may be that services regularly face fluctuations in demand and supply [1]. Although STAT is designed around the concept of balancing demand with supply, it cannot control for a long and unexpected reduction in supply or sudden, excessive increase in demand. Observations of site level data provide preliminary support for this hypothesis. For example, during a long period of absence due to illness of a staff member, there may be no way for a service to continue to provide the required number of new appointments to keep up with demand, gradually increasing the time to the next available assessment appointment. There are strategies that can be used to mitigate this effect, such as maintaining a contingency in budgets to cover unexpected leave or planning ahead for known disruptions. However, it is likely to be necessary to accept that there may need to be a periodic “reset”, in which there is a short-term focus on reducing excess waiting time that may be re-emerging, checking supply and demand calculations and making any necessary alterations to scheduling to ensure the supply of new assessment appointments in sufficient to balance demand. Although the need to monitor the balance between demand and new appointments was discussed during implementation, the level of adherence to this aspect of STAT after project support was withdrawn is not known. Having trigger points for discussion when times to the next available appointment reach certain thresholds and ensuring that contingency plans are in place are likely to play an important role in sustaining gains made by implementing STAT.

Diminishing effects over time may also be explained by the circumstances in which the intervention was implemented at the participating sites. The timing of the implementation of the intervention was dictated by the stepped wedge cluster randomised controlled trial design [16]. This meant that support from project officers was available only over a specified period. The motivation for introducing the intervention was also driven by the project team, rather than by a champion within the services [17]. Furthermore, in agreeing to be part of the project the service providers committed to continuing with the STAT model for the duration of the project, but made no commitment beyond the end of the stepped wedge trial period. It is therefore possible that the intervention had not been supported for long enough to be truly embedded into practice by the time support of the project team had been withdrawn.

A ‘real world’ setting, outside of a research study, is likely to present different issues in relation to sustainability of STAT as an intervention to reduce waiting time. First, without an artificial timeline in which to conduct the process of backlog reduction, service providers have a much better opportunity to reduce the existing waiting list as much as possible before implementing STAT. Maintaining momentum for change over a longer period is more likely where the need for change is recognised as a priority from an organisational, executive and managerial perspective [17]. Internal champions and a highly motivated team with a shared goal to reduce waiting times are therefore likely to improve sustainability of the model. STAT is a complex intervention, requiring skills in understanding and analysing data, attention to principles of change management, and a new way of thinking about prioritisation [11, 18]. Leadership, drive and an appropriate skill set are therefore required for successful implementation and sustainability of the STAT model. The current evaluation suggests that benefits can be maintained over 12 months, but questions remain about whether sustainability could be further improved with additional support over a longer time-period.

Examination of data over a longer period resolved the increase in failure to attend rate in the post intervention period observed in the original study. This unexpected finding in the stepped wedge cluster RCT was in contrast to previous literature that suggests that reduced waiting times are likely to be associated with a reduction in the rate of people who do not attend appointments [19, 20]. However, over a 12-month follow-up this effect was no longer observed; conversely, the number of missed appointments was observed to reduce by 14% when measured over 12 months, although the effect was not statistically significant. One explanation for the failure to attend results in the original study is that short-term increases in failure to attend rates occurred due to service disruptions associated with the process of dealing with the backlog and changing the model of care. For example, less attention may have been given to the appointment reminder systems that were in place at the participating services during this period. Once the new processes had been established, attention could return to standard work associated with smooth running of the clinic, and shorter wait times began to contribute to overall reductions in non-attendance.

This was a pragmatic evaluation of data routinely collected over a 12-month period following implementation of an intervention within a stepped wedge cluster randomised controlled trial. Data were collected retrospectively and limited to variables that were routinely collected by the participating sites. Some verification of data was possible through cross-referencing multiple data sources (such as clinician diaries with referral data) but limitations of retrospective data, such as the possibility of inaccurate or incomplete records, must be considered in interpretation of findings. Site level observations presented must also be treated with caution. The study was not powered for site by site analysis, and the data presented are post-hoc. However, these are included to illustrate the level of variability between services. Further exploration of the causes of variation and predictors of success in implementing STAT are areas for further research.

We also acknowledge that the STAT model contains multiple components, including a short-term, targeted intervention to reduce the existing backlog and ongoing protection of time for new assessments to match typical service demand. It could be argued that the design of this study is lacking in capacity to provide any insights into the relative contributions of the different elements of the intervention, and could be enhanced by inclusion of statistical analysis of interaction effects with a moderator analysis. However, STAT intentionally brings together a combination of evidence-based interventions to reduce waiting time which are not intended to work alone. Short-term increased in activity can reduce backlogs but do not lead to lasting change; preserving time for new appointments at the rate of demand without addressing the backlog will stabilise waiting time but will not reduce it. STAT should therefore be seen as a package, rather than a series of concurrent interventions that can be evaluated in isolation.

## Conclusion

The STAT model reduced waiting time in community-based outpatient services. This 12-month follow up shows that a significant benefit in overall waiting time continues to be observed after 12 months, although some small reductions in the effect over time are apparent. One key benefit, the reduction in variability and more timely service to people who previously waited the longest, appears to be sustained over 12 months. STAT is an effective strategy for reducing waiting time in these services, but is likely to require ongoing monitoring from champions within the service, including associated contingency planning and a willingness to, periodically, ‘reset’ the system after unexpected disruptions, to be fully sustainable over the long term.

## Data Availability

The datasets generated and/or analysed during the current study are not publicly available due to the conditions of ethical approval provided for the study, but are available from the corresponding author on reasonable request.

## Abbreviations

STAT: Specific Timely Appointments for Triage
IRR: Incident rate ratio
IQR: Interquartile range
